# Abstract withdrawn

**DOI:** 10.1186/1753-6561-5-S8-P90

**Published:** 2011-11-22

## 

Abstract withdrawn

